# Screening for the Efficacy of Botanicals and Soaps in Controlling the Banana Aphid *Pentalonia nigronervosa* (Hemiptera: Aphididae) Under Laboratory and Screenhouse Conditions

**DOI:** 10.3390/insects17010023

**Published:** 2025-12-23

**Authors:** Geofrey Ogwal, Peter Wasswa, Walter Ocimati, Bonaventure Aman Omondi, Anthony Fredrick Tazuba, Michael Hilary Otim, Guy Blomme

**Affiliations:** 1 Bioversity International, Kampala P.O. Box 24384, Uganda; ogwalgeff1996@gmail.com (G.O.); tazubatony@gmail.com (A.F.T.); 2 Department of Crop Science and Horticulture, Makerere University, Kampala P.O. Box 7062, Uganda; peterwasswa648@gmail.com; 3 Bioversity International, Cotonou 08 BP 0932, Benin; b.a.omondi@cgiar.org; 4 National Agricultural Research Organisation, National Crops Resources Research Institute—Namulonge, Kampala P.O. Box 7084, Uganda; motim9405@gmail.com; 5 Bioversity International, c/o ILRI, Addis Ababa P.O. Box 5689, Ethiopia; g.blomme@cgiar.org

**Keywords:** banana bunchy top virus, biopesticides, crop aphids, economic feasibility, insecticidal soaps

## Abstract

Controlling banana aphid (*Pentalonia nigronervosa*) vectors of banana bunchy-top disease is crucial for integrated disease management. Synthetic insecticides, though effective in aphid control, pose health and environmental risks. Biorational pesticides (including botanicals and soaps) are safer alternatives. This study screened four fresh and fermented aqueous plant extracts (chili pepper, garlic, neem, and peppermint) and three soaps (bathing, laundry bar, and liquid soap) singly or in mixtures against banana aphids through in vitro and in vivo experiments. Aphid-inoculated banana leaf-blade disks were used for the in vitro studies over 96 h. Potted plantlets were used for the in vivo studies over eight weeks. Applying these preparations resulted in significant (*p* ≤ 0.05) aphid mortality (28.6 to 100%) compared to the water control (3.3%) at 96 h post-spray (hps). In vitro, a single application of nimbecidine^®^ (Azadirachtin 0.03%), garlic, chili-pepper botanicals, insecticidal, and bathing soap caused >50% mortality at 96 hps. Mixed applications of chili pepper, garlic-based, and neem botanicals with soap caused >70% mortality at 96 hps in vitro. Binary mixtures of neem oil, garlic, fermented garlic, or fermented peppermint with insecticidal soap, and neem oil with bar soap, were promising in vivo, reducing the aphid populations to <20/plant compared to the control, which had >200 aphids after week 8. However, these potential preparations incurred a higher cost (USD 1.30/banana mat) compared to the synthetic pesticide (USD 0.022/mat). Thus, biorational mixtures have a potential to effectively control banana aphids.

## Simple Summary

The banana aphid (*Pentalonia nigronervosa*) spreads banana bunchy-top disease (BBTD), an important viral disease of bananas. Controlling aphids is therefore an important component of the integrated management of BBTD. Plant extracts and oils (botanicals) and soaps offer a safer alternative to synthetic insecticides. We screened fresh and fermented botanicals (chili pepper, garlic, peppermint, and neem oil) and soaps (bathing, laundry bar, and liquid soap) singly or in mixtures against banana aphids. The botanicals and soaps were compared with water and commercial products (nimbecidine (Azadirachtin 0.03%), insecticidal soap and inorganic pesticide, and Acetamectin Force). Though not as effective as commercial products, both the botanicals and soaps greatly reduced aphid populations in the laboratory. Single applications of nimbecidine^®^, garlic, chili-pepper botanicals, insecticidal, and bathing soap reduced aphids’ population by over 50% mortality at 96 hps, while mixtures of chili, garlic, and neem oil with soap caused over 70% mortality. On potted plants, binary mixtures of neem oil and botanicals (fermented and unfermented) with insecticidal soap, and neem oil with bar soap, reduced aphid populations to less than 20 per plant compared to 200 aphids for plants treated with water after week 8. Thus, these botanicals and soaps have the potential to contribute to integrated BBTD management.

## 1. Introduction

Banana bunchy top disease (BBTD), caused by the banana bunchy top virus (BBTV), is a significant threat to banana (*Musa* spp.) production worldwide, causing an up to 90–100% yield loss within one to two growing seasons if not effectively controlled [1]. In Sub-Saharan Africa, the disease is widespread across areas where bananas are cultivated [2]. In Uganda, BBTD was first reported in 2020 [3]. A more recent study [4] showed BBTD to be widespread, causing severe yield losses in northwestern Uganda and border regions of Kasese district in western Uganda. More recent outbreaks have also been reported in different locations in Tanzania [5,6]. Disease prediction maps [4,7] have shown key Ugandan banana-producing zones, especially in the Lake Victoria basin, to be very prone to BBTD. The disease is thus exerting increasing pressure on East and Central African banana production, since it is difficult to control once established, especially in smallholder farm settings [1,3].

BBTD is spread predominantly through infected planting materials and the BBTV vector the banana aphid *Pentalonia nigronervosa* Coquerel (Hemiptera: Aphididae) [8,9,10]. The aphid is an important banana pest where BBTD exists but, on its own, causes little economic damage. It is present in all banana-growing and -production regions worldwide [11,12]. The banana aphid can cause both direct and indirect damage [13]. The direct albeit minor injury occurs through sucking the phloem contents of all banana pseudostem segments [13,14]. Indirect but high economic damage occurs through the spread of BBTV [15]. The banana aphid acquires BBTV in a circulative, non-propagative manner and spreads it persistently [16,17]. This means BBTV circulates within the aphid’s body but without replication and can be retained and transmitted over a long period, often lasting for the aphid’s entire lifespan. Through dispersal of winged forms (alates) of *P. nigronervosa*, the virus can thus be spread to new, healthy host plants [10,16]. The development of alates could be triggered by environmental factors, changes in host suitability (due to disease, aging, or environmental stress), and an increase in numbers beyond a population threshold [16]. Keeping aphid numbers/colonies below a population threshold that minimizes triggering the development of winged individuals is one of the recommended practices for BBTD control [8]. Any intervention that reduces aphid population size is thus likely to result in fewer winged individuals, thereby limiting disease spread.

BBTD management relies on both cultural and chemical approaches. Cultural methods include using clean planting materials and rogueing diseased mats (i.e., cluster of plants emerging from a single underground rhizome) [18,19]. Chemical methods focus on controlling the vectors through pesticide application [20]. Efforts to control aphid vector populations using pesticides have relied on both contact and systemic pesticides, such as diazinon and imidacloprid [16,20]. Robson et al. [20] reported that aphid mortality following imidacloprid treatment was dose dependent. However, some insecticides have been discouraged and banned for use [21,22] due to their potential hazards to non-target insects, animals, beneficial organisms, and human health [23].

Biorational pesticides, such as botanical insecticides and insecticidal soaps, could offer an alternative to chemical pesticides for controlling banana aphids. Biorational pesticides are derived from biodegradable natural materials or products of metabolism of microbes, plants, animals, and minerals [23,24]. Most biorationals are relatively safe due to their target specificity to insects, low toxicity to animals, easy degradation, short residual effects, and low persistence on plants [25,26,27]. Botanical insecticides are derived from plants with insecticidal properties [28,29]. Meanwhile, insecticidal soaps are derived from plant oils that have been specifically selected and formulated for their insecticidal action [30].

Many botanical insecticides have been evaluated and shown to be effective against crop-infesting aphids and other insect pests [31]. For instance, botanical insecticides have been used to control aphids in crops such as field peas (*Pisum sativum*), kale (*Brassica oleracea*) [32], rapeseed (*Brassica napus*) [33], and giant rose (*Rosa* sp.) [34]. Some common botanical biorationals reported to effectively control aphids include chili pepper (*Capsicum annuum*), garlic (*Allium sativum*), hot pepper (*Capsicum frutescens*), Mexican sunflower (*Tithonia diversifolia*), neem (*Azadirachta indica*), and onions (*Allium cepa*) [28,32,35]. Some of these botanicals, for example, neem and wild garlic, were reported to control aphids when fermented [36]. These species of plants are abundant in most areas around homesteads [37]. Botanicals control insects through their toxic, repellent, anti-feedant, inhibitory, protein denaturation properties and ovipositional and physiological activities [38,39]. These studies show that botanicals could be integrated in vector control for BBTD management. To date, there are few studies on the use and efficacy of botanicals as a viable option for controlling the banana aphid. For example, Baloch et al. [40] reported that neem oil at 50 g/L controlled banana aphids in field studies, with a maximum reduction occurring seven days after spraying. Yongyue et al. [41] reported low toxicity of the castor bean (*Ricinus communis*), common lantana (*Lantana camara*), nerium oleander (*Nerium indicum*), and tomato (*Solanum lycopersicum*) extracts to *P. nigronervosa* in the laboratory. Thus, the efficacy of botanicals vary among plant species, justifying the need for their screening against target aphid species prior to their use in pest and disease management.

Some registered soaps (potassium salts of fatty acids) have been used as pesticides [42,43,44]. For instance, aphids in crops such as sorghum [45] and soybean [46] were controlled with insecticidal soaps. Dish-washing soap and liquid soap were reported to control aphids on garden peas [47] and rapeseed [35], respectively. Soap’s mode of action depends on direct contact with the insect. Upon contact, soaps penetrate the insect’s cuticle and disrupt cell membranes, thus causing insect desiccation, cell leakage, and death [26,46]. However, not all soaps produce or exhibit the same effect or efficacy. For instance, a study on the use and efficacy of soap for banana aphid control showed a 2% fish oil rosin soap to be least effective against the banana aphid due to its inherent low lethal action as compared with synthetic insecticides [48]. The authors recommend assessing a broad range of soaps, including readily available laundry soaps, for banana aphid control.

Some studies have reported that mixtures of botanical extracts improve control of insect pests including aphids [49,50,51,52,53]. Though synergistic effects have been claimed, the improved efficacy of biorationals in these studies look to be primarily due to the higher concentrations of the insecticidal compounds in the mixtures. For example, mixtures of chili pepper and garlic achieved better control of cowpea (*Vigna unguiculata*) insect pests resulting in higher yields [54]. Nzanza and Mashela [35] reported that neem extract combinations with other botanical extracts such as wild garlic cause more pest mortality. Mixtures of fermented coriander (*Coriandram sativam*) leaves, ginger (*Zingiber officinale*) rhizome, and sweet flag (*Acorus calamus*) rhizome achieved high tobacco budworm (*Spodoptera litura*) mortality [55].

Mixtures of botanical plant extracts with other organic and inorganic products have also been evaluated [27,56,57]. However, only a few studies have focused on mixing (combining) botanical extracts with soap. For instance, Megersa [31] added 1% powdered soap to Endod (*Phytolacca dodecandra*), garlic, and neem as a spreading agent soon after extraction to control pea aphids (*Acyrthosiphon pisum*). To break liquid surface tension, soaps were added to plant extracts in small quantities as surfactants (or emulsifiers) during crude extract preparation [27,58].

These studies show that botanicals and soaps are promising biorational alternatives for use in insect pest and disease vector management. However, their mixtures have not been evaluated against banana aphids. The aim of this study was to evaluate the efficacy and economic viability of non-fermented and fermented botanicals singly or in binary mixtures with soap in controlling banana aphids in vitro and in vivo.

## 2. Materials and Methods

### 2.1. Aphid Rearing

**Figure 1:**
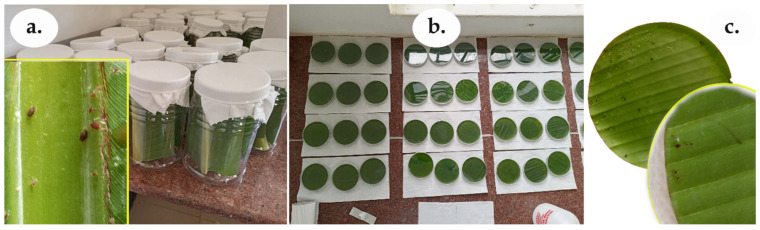
Aphid mass rearing and experimental setup. (**a**) Banana aphid mass rearing in plastic jars (inset are aphids of different sizes on a leaf portion in the jars); (**b**) leaf disk assay showing the Petri dishes (experimental units); (**c**) leaf disks with aphids in a Petri dish.

*Pentalonia nigronervosa* aphids were collected from banana plants in a garden at the National Agricultural Research Laboratories (NARL), Wakiso, Uganda (0.402364 °N, 32.527551 °E), using a soft number 3 nylon hairbrush and placed in 800 mL transparent plastic jars. The aphids were multiplied and maintained in vitro on ~5 × 12 cm^2^ fresh, midrib-containing banana-leaf sections in other plastic jars (Figure 1a) at room temperature (28 ± 2 °C), a relative humidity (RH) of 63 ± 3%, and a photoperiod of 12 light and 12 dark hours. No agar or water was added to maintain or lengthen the freshness of the leaves. The deteriorated/wilted leaves were replaced with fresh leaf tissues collected from the field at a weekly interval. Representative specimens of aphid progenies were examined under a microscope and confirmed as *P*. *nigronervosa* based on morphometric attributes [59].

### 2.2. Research Material and Preparation

The botanicals—chili pepper (*C. annuum*), garlic (*A. sativum*), neem (*A. indica*), and peppermint (*Mentha piperita*)—were selected based on their reported insecticidal efficacy against aphids in other crops and their local availability [36,37]. Stocks of aqueous extracts of chili pepper, garlic, and peppermint were prepared in the laboratory according to protocols by Iqbal et al. [60] and Kokwane [35]. Herein, 400 g of each fresh raw plant part (garlic bulbs, chili-pepper fruit, and peppermint leaves) were weighed using an electronic compact balance (A/B series analytical electronic scales, Beijing KWF Sci-Tech Development Co., Ltd., Rm 1904, Building No. 2, International Venture, Park No. 1 Shangdi, Haidian District, Beijing, China) and chopped into small pieces using a knife. For each species, the chopped plant part was then separately ground in an electronic blender (Philips, model HR2058) and dissolved in 1 L (1000 mL) of hot distilled water (≈80 °C) to make a 40% (*w*/*v*) stock solution. Hot water was used to increase extraction efficiency. The resultant solution was shaken by hand for about 10 s and left to stand for at least 24 h. The supernatant was first filtered using a nylon cloth (pore size about 0.125 mm) and thereafter filtered again using filter paper (Whatman No. 1) to achieve each botanical extract’s maximum refined form. The stocks were stored as a standard stock solution at 4 °C in 1 L glass jars (Duran^®^ borosilcate glass jars, DWK Life Sciences GmbH—Otto-Schott-Str. 21, 97877 Wertheim, Germany) bottle made of borosilicate glass 3.3) until use. Fresh stock solutions were prepared at monthly intervals. The storage glass jar lids were sealed using cling film to prevent any possibility of active-compound escape. Neem oil was prepared by cold-pressing the seeds [61], while a commercial-grade biopesticide nimbecidine (Azadirachtin 0.03%) was used as a botanical control.

The soap stocks used included bathing soap (Geisha brand with aloe vera scent), laundry bar soap (White Star brand with lemon scent), and liquid soap (Clinisafe brand) (Table 1). Stocks of both bathing and laundry bar soaps were prepared by weighing 10 g of each soap, chopping them into small pieces using a knife, and separately dissolving them in 900 mL of hot distilled water (≈80 °C), and topped to the 1 L mark to make a 1% (*w*/*v*) solution. A 1% (*v*/*v*) stock of liquid soap solution was prepared by diluting 10 mL of a ready-made liquid soap (Table 1) in 990 mL of distilled water. The resultant stock solutions were left to completely dissolve for 24 h and stored at room temperature until use. A commercial-grade liquid insecticidal soap (Neudosan) was used as the control soap (Table 1).

The stock solutions of chili pepper, garlic, peppermint, bathing soap, laundry soap, and liquid soap were used at the highest concentration (C6) (Table 2). The stocks were each diluted by mixing with distilled water to produce five lower concentrations, C1 to C5 (Table 2). Distilled water alone was used as the negative control treatment.

### 2.3. Preparation of Fermented Plant Extracts

The fermented plant extracts were prepared according to modified protocols by Kyan et al. [62], Nzanza and Mashela [36], and Khadem et al. [63]. The botanicals—chili pepper, garlic, and peppermint—were prepared at 40% (*w*/*v*) stock as described previously but not filtered. After 24 h, the unfiltered stocks were mixed with a carbohydrate mixture and the baker’s yeast *Saccaromycetes cerevisiae* (saf-levure^®^). The carbohydrate mixture was prepared in 50 mL falcon tubes at a ratio of 30 g:10 g:20 mL of brown sugar (containing sucrose), glucose, and warm distilled water (≈50 °C), respectively. Dry yeast was activated by dissolving 2 g in 10 mL of lukewarm distilled water (≈38 °C) in a separate 50 mL falcon tube, which was left to stand for 10 min and stirred well. A total of 50 mL of the mixture containing the carbohydrate mix, plus 10 mL of the mixture containing yeast were added to each half liter volume of plant-extract stock filled to the mark (with headspace) in an airtight glass jar. The bottles containing the fermentation mixtures were kept at room temperature (26 ± 2 °C) for 21 days. The carbohydrate mixture was used to supply energy needed for the yeast, mimicking the main components of molasses used in fermentation by microbes.

Carbon dioxide gas, a by-product of fermentation, was released once every week from the fermenting mixture by opening the lid of each glass jar [63]. After incubation, the fermented extracts were filtered through a fine cloth, and then subsequently through a 15 cm diameter filter paper (Whatman No. 1). The final pH values of the fermented preparations were measured as follows: fermented garlic (4.18), fermented chili pepper (4.04), fermented peppermint (4.40), and the fermented carbohydrate–yeast mixture with distilled water only (3.80), in contrast to distilled water (7.0). Each of the filtered stock solutions (40%) of fermented chili pepper, garlic, and peppermint were used as formulated. The fermented carbohydrate–yeast mixture with water only was used as the negative control for fermented botanical extracts.

### 2.4. Preparation of Plant–Soap Mixtures

All non-fermented botanicals (neem oil, nimbecidine, and filtrates of chili pepper, garlic, and peppermint) and fermented botanicals (chili pepper, garlic, and peppermint) were each mixed with the two best and/or cheap soaps (insecticidal soap and/or laundry bar soap) (Table 1) in a ratio of 1:1 (*v*/*v*) of botanical/soap. The highest concentrations derived from a concentration–mortality response in single preparation applications, except for neem oil and insecticidal soap, were the optimal recommended concentrations used. The filtered fermented botanicals were used as formulated.

### 2.5. Bioassays: Assessment of Botanicals and/or Soap In Vitro

The bioassays were conducted based on modified protocols of the Insecticides Resistance Action Committee (IRAC) through the detached leaf-disk assay method for aphids [64]. Modifications on the protocol included using 1.5% (*w*/*v*) agar instead of 1% agar, fewer (10) individual aphids instead of 15 to 20 aphids, and abaxial leaves instead of the adaxial leaves. The 1.5% (*w*/*v*) agar solution was prepared by dissolving 15 g of bacteriological agar (Agar Agar powder No. 1, Loba Chemie Pvt Ltd.—Jehangir Villa, 107 Woodhouse Road, Colaba, Mumbai 400005, Maharashtra, India) in 1 L of distilled water. The suspension was then autoclaved at 121 °C and 15 pounds per square inch (psi) for 15 min.

After autoclaving, the agar solution was gently turned upside down to obtain a uniform mixture. After cooling to about 45 °C, the solution was dispensed in 90 mm diameter Petri dishes to a depth of about 0.8–0.9 cm in a laminar floor hood and allowed to cool and set overnight. Excess condensed water was wiped off the top inner sides of the transparent lids using a sterile kitchen towel. Fresh, green, disease-free young leaves with a softer lamina (leaf blade) were collected from the banana fields at NARL and surface sterilized with 70% ethanol to minimize fungal growth on cut leaf disks by wiping using kitchen towels or cotton wool. The leaves were then left for about 30 min to allow for evaporation of ethanol traces before cutting.

Circular banana leaf disks with a 90 mm diameter were carefully cut from whole East African highland banana (*Musa* AAA-EAHB genome) leaves of mixed cultivars using a circular glass Petri-dish cover template and a sterile surgical blade to avoid tearing through the leaf blade. Leaf blade portions near the leaf margin (marginal veins) of small leaves from young banana plants (less than 1 m in length), without any physical, physiological, or pathological defects, were used [20]. The leaf disks from different leaves and plants were randomized to different concentrations to minimize variations within the leaf disks. The circular leaf disks were carefully placed onto the agar media, with the abaxial (lower) surface down (Figure 1c) to keep the leaf disk in place and fresh during the experiments.

Ten adult banana aphids were randomly introduced to each of the Petri dishes using a soft number 1 fine nylon hairbrush (Figure 1c). The aphids were allowed to settle on the leaf disks overnight before applying the treatments. The aphids in the Petri dishes were each sprayed once with the different treatments in their respective concentrations and/or mixtures with 4 mist shower shots (≈2 mL volume), using separate 1 L multipurpose, handheld sprayers. The shower shots were performed to uniformly cover the Petri dish area [56] and to ensure aphid contact with the treatment solution while minimizing drowning.

### 2.6. Experimental Design for the In Vitro Experiments

The in vitro experiments were placed in a completely randomized design (CRD). The treatments (Table 1) were replicated six times. Each experimental unit comprised a 90 mm plastic Petri dish containing a circular banana leaf disk of 90 mm in diameter (Figure 1b). For single (un-mixed) applications of non-fermented botanicals or soaps, all six concentrations (i.e., C6, C5, C4, C3, C2, and C1) (Table 2) were used. For single applications of fermented botanicals, the stock concentration (filtrate, i.e., 40%) was used.

For binary treatment mixtures of non-fermented botanicals with soap, the most potent concentration, 40% (C6), causing higher aphid mortality, was used, while for mixtures of fermented botanicals with soap, the stock filtrate was used. Distilled water served as the negative control, while the systemic pesticide Acetamectin Force*^®^* (0.15% *v*/*v*) served as the positive control. The experiments were conducted over 4 days (96 h) at a room temperature, RH, and photoperiod of 29 ± 4 °C, 65 ± 5%, and 12:12 h light–dark, respectively. Only adult aphids selected from the laboratory-reared aphid colonies were used for the various bioassays.

After administering the treatments, the numbers of living and dead aphids were recorded daily (24 h intervals between 15:00 and 18:00 h EAT) over a four-day period. The aphids (especially nymphs) were counted with the help of a 10× magnifying hand lens. The aphids were considered dead if they failed to respond to a gentle prod on the body using a number 1 soft and fine nylon hairbrush. Dead aphids were removed each day after being counted and recorded.

### 2.7. Experimental Design for the In Vivo Experiments

**Figure 2:**
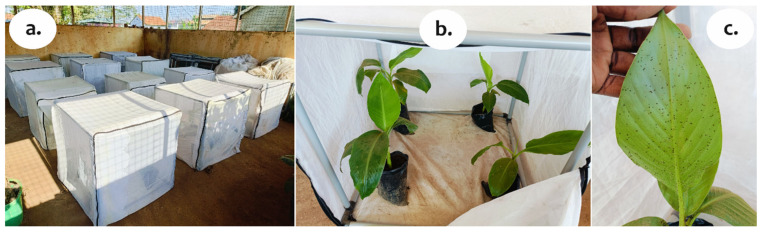
In vivo experimental setup. (**a**) Insect-proof cages; (**b**) Experimental units (potted plants in a cage); (**c**) Infested potted plant leaf in the screenhouse.

The in vivo experiments were laid out in four replicates in a CRD. A total of 12 treatments were evaluated (Figure 2a), informed by analysis of the in vitro experiments. The treatments included spraying potted plants with mixtures of chili pepper, fermented chili pepper, garlic, fermented garlic, peppermint, fermented peppermint, and neem oil with insecticidal soap, and mixtures of fermented chili pepper, garlic, neem oil, and nimbecidine with bar soap. Distilled water was included as a negative control.

Visibly healthy micro-propagated banana plantlets of the cultivar (cv.) ‘Mpologoma’ (*Musa* AAA-EAHB genome) were obtained from a private tissue culture laboratory (Agro Genetic Technologies Laboratories, Buloba, Uganda). The plantlets were transferred to larger polyethylene potting bags (20 cm diameter and 24 cm depth, perforated to avoid water logging), filled to a 3 L capacity with a mixture of sterilized forest topsoil, composted chicken manure, and sand in the ratio 3:1:1, respectively. The plantlets were transferred into insect-proof cages (80 cm long × 70 cm wide × 75 cm high with covering of insect netting cloth of about 0.125 mm mesh size) (Figure 2a) for in vivo evaluations.

Each experimental unit comprised four potted plants in a cage (Figure 2b). The plantlets were allowed to acclimatize and grow to about 20–30 cm tall (with at least 3–4 fully developed leaves) in the insect-proof cages within a screenhouse before aphid infestation. Each cage cloth was fitted with a zipped door to allow for manipulation. Watering was performed weekly directly into the soil substrate at soil level rather than to foliage to minimize washing the aphids off the plants. No fertilizer was applied to the plants during the study period, but soils in the potting bags were topped up with the soil mixture (described above) at the onset of first sprays to replenish soil carried off during watering.

Mixed aphid populations (nymphs and adults) from the laboratory-reared colonies were used for infestation in the screenhouse. Each plantlet was infested with 20 aphids on the youngest, partially unfolded leaf (cigar leaf). Efforts were made to randomize aphids of different ages/sizes equally across the plants. Aphid population build-up (as seen in Figure 2c) was allowed over a four-week period before treatment application. Plants were re-randomized between the cages to ensure that plants with the same abundance of aphids were uniformly spread between the cages at treatment application. This was performed according to the aphid abundance score modified from Biale et al. [65]. For each of the 12 treatments, the plantlets with visible aphid colonies were sprayed with 100 mL solution to wetness at two-week spray intervals over a two-month period (i.e., 4 sprays). Separate 1 L multipurpose handheld sprayers were used for each treatment. The sprayers provided a misty spray, which avoided physically dislodging the aphids. The treatments were only administered through spraying in the evening, and the sprayers were thoroughly rinsed afterwards.

The number of living aphids was recorded at onset before spray application. Aphids were counted with the help of a 10× magnification hand-lens. The plants were then monitored for aphid population dynamics weekly for eight more weeks after first sprays. A thick, hard, white paper was placed at the base of each plant to collect dead aphids by gently tapping the plants. The dead aphids physically seen from the plants were removed before initiating the next spray. All plants were monitored for symptoms of phytotoxicity due to the treatments.

### 2.8. Cost and Risk Associated with the Preparations

As a measure of the ease of access and use, the costs and risks associated with the different biorationals and controls were also assessed. This was based on market prices, the effective application rates, and researchers’ experiences in their preparation and application. The market costs of all biorational treatments were obtained through local purchases in Uganda.

### 2.9. Statistical Analysis

Data analysis was performed in RStudio v2024.4.2.764 [66] using R programming language v4.4.1 [67]. For in vitro bioassays, the percentage of cumulative daily mortality of the aphids was calculated as

$$\mathrm{Cumulative}\;\mathrm{mortality}\;(\%)=\frac{\mathrm{Cumulative}\;\mathrm{number}\;\mathrm{of}\;\mathrm{dead}\;\mathrm{aphids}}{\mathrm{Number}\;\mathrm{of}\;\mathrm{living}\;\mathrm{aphids}+\mathrm{cumulative}\;\mathrm{number}\;\mathrm{of}\;\mathrm{dead}\;\mathrm{aphids}}\;\times \;100$$

Prior to analysis, mortality data were checked for normality using the Shapiro–Wilk test and for skewness within a repeated-measures mixed-effects framework. The model residuals met the assumptions of approximate normality, homogeneity of variances, and absence of strong skew; therefore, no data transformation was applied. A repeated-measures ANOVA was then performed, considering treatments and concentrations as fixed factors, post-spray hours as the repeated-measures factor, and replications as random factors. The significant individual factors and/or factor interactions were considered for post hoc tests. Means were separated using Tukey’s honest significant difference (HSD) at 5% significance.

Probit regression curves were generated for dose–mortality responses. The doses were log_10_-transformed in R, while the probit of probability mortality (probit units) was calculated using the formula:

$$Probit\;\left(mortality\right)\;units=\left[qnorm\left(\frac{\mathrm{mean}\;\mathrm{percentage}\;\mathrm{mortality}}{100}\right)\right]+5$$

where “qnorm” is the standard normal quantile function in R, and the “+ 5” is an adjustment value to avoid negative probit values.

The mean lethal concentration 50 (LC_50_) was estimated using the “LC_probit” function in the ecotox package in R.

For in vivo data, the proportion of living aphids was calculated as

$$\mathrm{Living}\;\mathrm{aphid}\;\mathrm{proportion}\;(\%)=\left(\frac{\mathrm{Number}\;\mathrm{of}\;\mathrm{living}\;\mathrm{aphids}\;\mathrm{at}\;\mathrm{week}\;\mathrm{after}\;\mathrm{first}\;\mathrm{spray}}{\mathrm{Number}\;\mathrm{of}\;\mathrm{living}\;\mathrm{aphids}\;\mathrm{at}\;\mathrm{onset}\;\mathrm{before}\;\mathrm{spray}}\;\times \;100\right)$$

The proportion of aphid mortality was calculated as

$$\mathrm{Mortality}\;\mathrm{proportion}\;(\%)=100-\left(\frac{\mathrm{Number}\;\mathrm{of}\;\mathrm{living}\;\mathrm{aphids}\;\mathrm{at}\;\mathrm{day}\;\mathrm{after}\;\mathrm{spray}}{\mathrm{Number}\;\mathrm{of}\;\mathrm{living}\;\mathrm{aphids}\;\mathrm{at}\;\mathrm{onset}\;\mathrm{before}\;\mathrm{spray}}\;\times \;100\right)$$

The model residuals for percentage of living aphids and aphid mortality assumed a normal distribution; thus, transformations were not necessary.

In a repeated-measures ANOVA, the treatment (type of biorationals) was considered as the fixed factor and replication as the random factor. “Weeks” was a repeated-measures factor for the number and percentage of living adults that were monitored weekly during 8 weeks after the first spray. “Spray weeks” was a repeated-measures factor for aphid mortality percentage that was recorded at four bi-weekly points. Significant factors were examined in post hoc tests, as for the in vitro analysis.

For the cost and risk assessment, market costs of the chemical pesticide and biorationals were used to compute the cost of 1 L of the most effective dosage of pesticide solution. The cost of the solution volume required to spray one banana mat containing four plants was computed. The ease of preparing biorationals and chemical alternatives was rated on a subjective scale of 0 (super easy) to 5 (very difficult). The possible human risk at the recommended dosage of pesticide was rated on a scale of 0 (no risk) to 5 (very high risk). Labor (for spraying insecticides) and transport costs were excluded from the assessments. An exchange rate of 3700 UGX per US dollar (USD) was used.

## 3. Results

### 3.1. In Vitro Evaluation of Botanicals and Soaps

#### 3.1.1. Aphid Mortality After Single Application of Non-Fermented or Fermented Botanicals and Soaps

**Figure 3:**
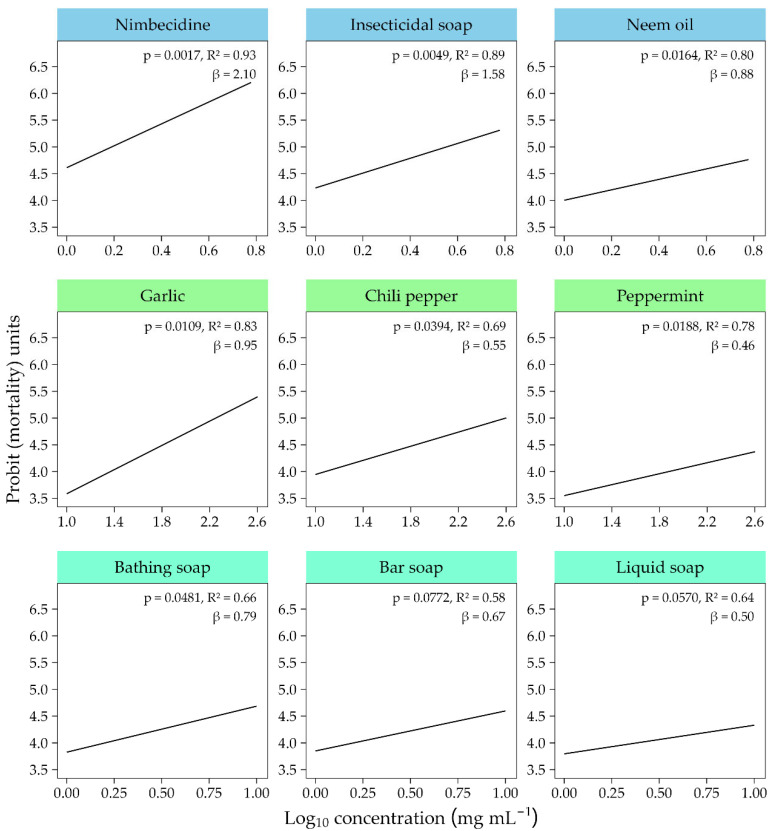
Probit regression dose–response curves showing the effect of non-fermented botanical or soap concentrations on cumulative aphid mortality at 96 hps.

There were highly significant differences in aphid mortality between the non-fermented biorational treatments (F_8,1060_ = 189.0, *p* < 0.001), biorational concentrations (F_5,1060_ = 144.3, *p* < 0.001), and hours post-spray (hps) application (F_3,1060_ = 107.9, *p* < 0.001) (Supplementary Materials Table S1). The two-way interactions in aphid mortality between the treatments and concentrations was also highly significant (F_40,1060_ = 5.3, *p* < 0.001). Mortality significantly increased with concentration from C1 to C6 (R^2^ = 0.78 to 0.97; Figure 3) and continued to rise at higher concentrations (Supplementary Materials Figure S1). The probit probability of aphid mortality was highest for nimbecidine, insecticidal soap, and garlic, which exhibited the steepest concentration–mortality slopes, indicating a stronger dose-dependent response, and lowest for peppermint and liquid soap (Figure 3).

The mean LC_50_ was determined for each of the treatments. The estimates of the LC_50_ values for botanicals were lowest for nimbecidine and highest for peppermint, while for soap solutions, they were lowest for insecticidal soap and highest for liquid soap (Table 3). Nimbecidine and garlic showed the highest slope values (an indicator of steepness) among botanicals, while insecticidal soap and bathing soap showed the highest slopes among soaps (Table 3).

At the highest concentration, C6, aphid mortality was significantly different between all non-fermented or fermented botanicals or soap treatments (F_14,280_ = 127.4, *p* < 0.001) and hps (F_3,280_ = 103.0, *p* < 0.001) (Supplementary Table S1). Mortality increased with time from 24 to 96 hps. At 24 hps, the mean aphid mortality was higher for the Acetamectin Force positive control (98.4 ± 0.4%) compared to 6.7 ± 1.4 to 87.2 ± 4.1% for the biorationals. Mortality of aphids treated with nimbecidine was similar to that of those treated with Acetamectin Force but was significantly higher than mortality due to insecticidal soap. Both nimbecidine and insecticidal soap showed higher mortalities (>50%) compared to other biorationals which had mortalities of less than 50%, though all biorationals (except for fermented peppermint and fermented garlic) had a significantly higher mortality than the water controls (Table 4).

Similar trends to treatments at 24 hps were observed for the treatments at 48 hps, with Acetamectin Force at a maximum (100 ± 0%). At 72 hps, aphid mortality due to biorational treatments ranged from 24.4 ± 4.2 to 89.6 ± 3.7% and was significantly highest (5% Tukey HSD) for non-fermented garlic, fermented chili pepper, and nimbecidine. Similar trends to those at 72 hps were observed for the treatments at 96 hps, with aphid mortality due to the biorationals varying between 28.6 ± 4.1 to 91.6 ± 3.1% (Table 4). No significant differences (5% Tukey HSD) occurred between fermented chili pepper, non-fermented garlic, and nimbecidine at 96 hps. The insecticidal soap was significantly superior to the other soaps (5% Tukey HSD).

#### 3.1.2. Aphid Mortality Due to Mixing Non-Fermented or Fermented Botanicals with Best Soaps

Significant differences in aphid mortality were observed due to the treatment mixtures (F_17,340_ = 95.9, *p* < 0.001) and hps (F_3,340_ = 86.0, *p* < 0.001) (Supplementary Materials Table S1). Mortality increased with time from 24 to 96 hps. At 24 hps, the mean aphid mortality was significantly higher (5% Tukey HSD) for Acetamectin Force (99.9 ± 0.1%) compared to 10.8 ± 3.8 to 85.4 ± 5.0% for the biorational mixtures. Mixtures of nimbecidine, garlic, neem oil, fermented peppermint, chili pepper, fermented chili pepper, or fermented garlic with insecticidal soap, as well as nimbecidine with bar soap (71.0 ± 6.9 to 93.8 ± 2.0%) caused similar aphid mortality compared to the main positive control. Minimal aphid mortality of less than 40% was observed only with the fermented peppermint + bar soap treatment, which did not differ from the water control at 5% Tukey HSD (Table 5).

At 48 hps, aphid mortality was highest and at a maximum for AAcetamectin Force and nimbecidine + insecticidal soap (100 ± 0%) compared to 24.6 ± 6.2 to 73.4 ± 7.4% for other biorational mixtures. All biorational mixtures, except for fermented peppermint + bar soap, were significantly higher (5% Tukey HSD) than the water control. Similar trends to 48 hps were observed for the treatments at 72 and 96 hps, with garlic + insecticidal soap reaching maximum (100 ± 0%) at 72 hps, and fermented peppermint + bar soap causing significantly higher mortality than water control. Mortality increased with time from 24 to 96 hps, with water control mortality at less than 4% after 96 hps. Treatment mixtures causing greater than 50% average aphid mortality at all hps were advanced for the in vivo experiments. The main positive control (Acetamectin Force), nimbecidine (botanical control), and insecticidal soap (soap control) were not advanced for the in vivo experiments (Table 5).

### 3.2. In Vivo Evaluation of Selected Botanicals Mixed with Soap

#### 3.2.1. Aphid Population Dynamics Due to Mixtures of Biorationals with Soap

Significant differences (*p* < 0.001) were observed between the treatments in the number and proportion of living aphids, separate times of assessment (*p* < 0.001), and their interaction (*p* < 0.001) (Table 1; Supplementary Materials Tables S1 and S2). Except for the water control, in which the aphid population continuously increased, the number of living aphids fluctuated across weeks following each spray, dropping over the first week after each treatment application before rising again, albeit at different rates in the subsequent week (Table 6; Supplementary Materials Figure S2). Mixtures of neem oil, garlic, and fermented garlic with insecticidal soap and neem oil with bar soap displayed the best performance across the entire eight weeks, showing a general decline in aphid population with bi-weekly treatment applications. These were followed by mixtures of garlic and fermented chili pepper with insecticidal soap (Table 6; Supplementary Materials Figure S2). A similar trend to that for the number of living aphids was observed for the proportion of living aphids (Supplementary Materials Table S2).

There were spikes in aphid populations for the other biorationals, visible in the second week post-treatment. Mixtures of chili pepper and peppermint with insecticidal soap, and fermented chili pepper and nimbecidine with bar soap had a moderate efficacy during the eight weeks of evaluations. The aphid population in the water control increased exponentially, peaking at about 300% (~200 aphids) of the original population after six weeks (Table 6 and Supplementary Table S2).

Aphid mortality significantly varied (F_11,132_ = 111.2, *p* < 0.001) with the type of biorational treatment. Aphid population fluctuated across time after each spray event with biorational mixtures, except for neem oil + insecticidal soap and garlic + bar soap, for which populations kept decreasing. The aphid population in the water control always increased with time after spray (Supplementary Table S3). Aphid mortality for all biorational treatment mixtures was significantly higher (5% Tukey HSD) than for the water control after each spray time. Aphid mortality for the biorational mixtures ranged from 30.3 ± 7.8 to 80.4 ± 1.2% at first spray, increasing to 51.1 ± 22.4 to 97.8 ± 1.1% at the fourth spray. However, fermented peppermint + insecticidal soap showed a decline in the proportion of dead aphids from 87.8 ± 2.6% at the second spray to 48.5 ± 37.6% at the fourth spray.

Neem oil, fermented garlic, fermented chili pepper, and garlic, each mixed with insecticidal soap, showed the best efficacy across the entire spray period, with greater than 70% aphid mortality. Nevertheless, they were all statistically similar. Chili pepper + insecticidal soap, nimbecidine + bar soap, and neem oil + bar soap showed moderate efficacy, with more than 60% aphid mortality from the second to fourth sprays. Most of the mixtures evaluated showed low or no phytotoxicity (Supplementary Table S3).

#### 3.2.2. Cost and Risk Associated with the Preparation of Biorationals

The cost per liter of the working spray solutions of the single biorationals ranged from USD 0.014 (liquid soap) to USD 0.570 (fermented biorationals), while the cost of mixtures ranged from USD 0.074 (nimbecidine + bar soap) to USD 0.705 (mixtures of insecticidal soap with fermented chili pepper or fermented garlic or fermented peppermint) (Supplementary Table S4). Assuming 1.8 L of spray is needed per mat of four banana plants, the trends in cost of spray per mat were similar and ranged from USD 0.024 for liquid soap to USD 1.269 for the mixtures. In contrast, the cost of Acetamectin Force was USD 0.012 and USD 0.022 for the 1 L working solution and the 1.8 L solution needed to spray one mat, respectively. In terms of ease of preparation, neem-based treatments and all soap treatments were easy to prepare, while fermented products were difficult to prepare (thus labor-intensive). At the recommended dosage, neem oil-, garlic-, and peppermint-based biorationals were presumably less risky to humans. Acetamectin was rated poorly for ease of preparation and environmental risk due to its unpleasant pungency and poisonous effect to humans and the environment in general (Supplementary Table S4).

## 4. Discussion

This study evaluated the potential of biorationals to control the banana aphid *P. nigronervosa*, the main vector of BBTV, for possible inclusion into integrated pest and disease management (IPDM) strategies for BBTD. Compared to negative control, all the biorationals caused aphid mortality in vitro, with the highest cumulative mortality recorded at 96 h post-spray (hps). The highest number of aphids died within the first 24 hps, increasing cumulatively over time. These findings align with previous studies [37,58,60] and support the evidence that botanicals contain toxic compounds [38,39] that are typically more active within the first 24 hps [37]. The reduced rate of mortality beyond 24 hps can be attributed to the rapid degradation of the bioactive substances [31].

Aphid mortality, increasing with higher concentrations of non-fermented botanicals, was consistent with findings by Kayange et al. [37]. The comparable aphid mortality with non-fermented garlic at the highest concentration to nimbecidine at 96 hps suggests that it is highly effective at high dosages. However, from a practical and economic standpoint, sourcing or preparing such high-concentration solutions may be challenging for large-scale field application [37]. All biorationals outperformed the water control, especially from 72 to 96 hps, suggesting partial to substantial efficacy against banana aphids. The differences in aphid mortality across biorationals likely resulted from variations in their insecticidal activity due to differences in content, quality, and potency of active ingredients over time [68,69].

Fermented botanicals, particularly chili pepper and garlic, also demonstrated good insecticidal activity. This aligns with findings from Nzanza and Mashela [36] and Khadem et al. [63]. Fermentation modifies the chemical composition of bioactive substances present in botanicals [70,71], which may explain the reduced mortality observed with fermented vs. non-fermented garlic. However, it is still necessary to optimize fermentation conditions regarding materials used and time allowed for the fermentation process. Fermented chili pepper likely produces secondary metabolites—such as capsaicinoids (an alkaloid), flavonoids, glycosides, phenolic derivatives, and terpenoids—with stronger insecticidal effects [72,73]. The metabolites help protect plants from biotic stressors by acting as toxins, repellents, or natural enemy attractants [73]. Garlic fermentation, on the other hand, reduces allicin content, which is key to its toxicity [71]. Further studies are needed to identify and quantify these compounds in fermented versus non-fermented extracts.

Soaps applied singly also showed increased aphid mortality with increasing concentrations. This could be attributable to the disruption of insect cell membranes by fatty acids in soaps. Among all the soaps evaluated, bathing soap was the most comparable to insecticidal soap, indicating notable efficacy. Differences in efficacy across soaps can be linked to variations in their chemical composition and surfactant properties. Lower aphid mortality with insecticidal soap (63.4%± 4.8 at 72 hps) in this study compared to Kraiss and Cullen [46] [83.3% mortality of *Aphis glycines* with 2% soap applied] may be due to the lower concentration used (0.6%). When the concentration was increased to 1%, as in mixtures, banana aphid mortality rose to 96.7% (Supplementary Figure S1). Differences in banana aphid mortality compared to other aphid species could also be due to differences in their sensitivity to various biorationals or chemical insecticides.

Lower mortality with laundry bar and liquid soaps at a 1% concentration agrees with findings on *Trialeurodes vaporariorum* by Arias et al. [74] and reports by Curkovic [27]. However, increasing concentrations to 3–10% improved efficacy significantly. For instance, a 3% laundry bar soap solution and a 10% liquid soap solution resulted in 65.1% and 75.2% mortality, respectively, at 24 hps (Supplementary Figure S1). Similar results have been reported for *Dactylopius opuntiae* and cabbage aphids [35,75]. Application of viscous, high-concentration soap solutions may be improved by preheating to reduce clogging and enhance ease of spraying. Declining mortality beyond 24 hps is likely due to degradation of active ingredients and the need for the soap to remain in liquid form for maximum contact efficacy [31,57,76].

Mixtures of botanicals (e.g., nimbecidine, neem oil, garlic, fermented peppermint) with insecticidal soap demonstrated comparable mortality to Acetamectin Force. This is supported by Mwanauta et al. [77] and Siam and Abu-Elela [78], who also observed over 85% pest mortality using botanical–soap mixtures. These combinations improve the uniformity and persistence on plant surfaces, thereby enhancing contact with insects [54]. The increased effectiveness of soap–botanical mixtures may be due to synergistic effects from combining different modes of action [57,79]. Notably, neem oil, non-fermented garlic, fermented chili pepper, and fermented peppermint mixtures with insecticidal soap demonstrated high effectiveness. Mixtures involving bar soap were less effective than those with insecticidal soap, though they still performed well with botanicals like nimbecidine, neem oil, and fermented chili. Interestingly, the nimbecidine + bar soap mixture was less effective than nimbecidine alone, suggesting potential incompatibility or a dilution effect that might have reduced the mixture’s overall efficacy [57]. Studies exploring combinations of different plant extracts to harness potential additive or synergistic effects for better efficacy are lacking and recommended for banana aphid control.

In vivo evaluations showed that all soap–biorational mixtures significantly reduced aphid populations over eight weeks, consistent with in vitro findings, and demonstrating the value and effectiveness of combining biorationals with different modes of action [79]. Neem oil- and garlic-based mixtures with insecticidal soap were particularly effective against banana aphids, corroborating results with papaya mealybugs from Mwanauta et al. [77]. Slight population rebounds observed in the second week after sprays likely resulted from declining stability and degradation of active compounds upon exposure to environmental sunlight, moisture, and air [31]. This highlights the need for timely reapplication and alignment with periods of lower pest pressure. The rapid degradation rate of these compounds could also minimize environmental risks and harm to non-target organisms.

Peppermint mixtures showed inconsistent results, potentially due to their strong repellent effects which may have caused aphids to dislodge rather than die [80]. This study did not, however, determine the repellent effects of the botanicals. The difference in efficacy of neem and other botanicals across weeks may be influenced by variation in their composition and stability, which could have depended on plant material source, plant maturity, and extraction quality [31,81]. The use of market-sourced and backyard-grown materials may have limited the uniformity of results. Lower mortality observed with garlic + bar soap and fermented peppermint + insecticidal soap after the fourth spray (c.f. Supplementary Table S3) may be due to increased aphid reproduction or concealment within leaf sheaths [82].

Aphid populations in the water control consistently increased, indicating natality and population growth under no treatment. In contrast, the best-performing mixtures significantly reduced aphid populations, indicating their potential as sustainable aphid management tools. As previously observed by Walter [83] and Harris [57], the neem-based mixtures showed promise for use against banana aphids and in future biorational pesticide development. While some mixtures exhibited mild phytotoxicity (Supplementary Table S3), especially those with insecticidal soap [31], their overall efficacy indicates strong potential for integration into IPDM strategies. This can be further supported by the overall objective of not eradicating all aphids, but of maintaining aphid populations below the threshold that allows the development of winged individuals, especially in mats close to rogued symptomatic plants/mats.

The synthetic insecticide Acetamectin Force was the cheapest option but comparable to the soaps when used singly. In contrast, the botanicals and fermented or mixed formulations were more expensive and often labor-intensive to prepare, limiting their practicality on farms. The high costs of biorationals and effort-intensive nature of their preparation were attributed to higher concentrations used/required. Similar observations of the high cost of biopesticides and their limited on-farm adoption have been reported by Malinga and Laing [84] and Fusar Poli et al. [85]. In addition, most of the biorationals were sourced from urban markets, suggesting potential access limitations in rural environments. From an environmental perspective, all biorationals could presumably pose low to moderate risks at recommended dosages. Natural products like peppermint, garlic, and their fermented forms have been reported to have a low-risk profile [23]. Despite their higher cost and preparation demands, fermented formulations are often valued for their broad-spectrum activity, which can enhance pest control and delay resistance development [72]. Therefore, a trade-off between affordability, sustainability, and efficacy—driven by farmer objectives and production constraints—must be carefully considered when integrating biorationals into the IPDM of BBTD.

## 5. Conclusions and Recommendations

This study demonstrates the potential of eco-friendly, biorational products for controlling banana aphid populations. Mortality varied with biorational-extract concentration and evaluation time, with mixtures showing superior control. Binary mixtures of neem oil, garlic, or fermented garlic with insecticidal soap were particularly promising. These suggest a strong foundation for developing biorational formulations for banana aphid management. Effective pest control will require optimized mixture stability and precise application timing.

Effective control should aim to suppress aphid populations below the threshold that triggers the development of winged forms, and by limiting alate dispersal through their immobilization and/or death. Rather than relying on widespread applications to eliminate all aphids in a field, a pragmatic IPDM strategy could focus on targeted sprays/treatments—particularly on rogued infected plants and plants adjacent to them, i.e., asymptomatic plants and mats that might already be infected—to reduce the risk of further BBTD spread.

The feasibility of using biorationals to control banana aphids varies according to formulation, balancing cost, ease of preparation, and human safety. While simple soap-based solutions are affordable and eco-friendly, complex botanical mixtures may offer benefits within IPM frameworks despite logistical challenges. Integrating economic and practical considerations is key to sustainable adoption. Further field trials are needed to validate their real-world effectiveness and operational viability.

## Figures and Tables

**Table 1:** List of treatments (insecticides, botanicals, and soaps) used in the study.

Treatment	Source	Single Application Rate (g/L or mL/L)	Application Rate in Mixture (g/L or mL/L)
Chili pepper	Backyard gardens	100–400 g	400 g
Garlic and peppermint	Local market (LM)	100–400 g	400 g
Neem oil	NARL, Kawanda	1–6 mL	10 mL ^a^*
Nimbecidine^®^ (Positive control)	RealIPM, Uganda	1–6 mL	6 mL
Bathing soap and laundry bar soap	LM	1–10 g	10 g ^b^*
Liquid soap	Joint Medical Store, Uganda	1–10 mL	-
Neudosan insecticidal soap (Positive control)	RealIPM, Uganda	1–6 mL	10 mL
Acetamectin Force^®^ (Main positive control)	LM	1.5 mL	1.5 mL
Distilled water (Main negative control)	NARL	0 mL	0 mL
Fermented carbohydrate–yeast mixture with water only (Negative control)	This study	0 mL	0 mL

According to the World Health Organization (WHO) classification, nimbecidine^®^ (ai. Azadirachtin 0.03% + neem oil 90.57%) and Neudosan (ai. potassium salts of fatty acids 470 g/L (47%)) are categorized under class IV (i.e., safe), while Acetamectin Force^®^ (ai. Acetamiprid 3% + abamectin 1.8% EC) is categorized as class II (i.e., hazardous); treatments in bold were additionally fermented at the highest concentration (400 g/L); ^a^* application rate increased in mixtures due to lower mortalities observed. ^b^* Laundry and bathing soap were not significantly different (*p* > 0.05) in the single application experiments. As such only laundry bar soap, which was cheaper to access, was used. The best mixtures with greater than 50% mortality at all post-spray hours in vitro were advanced for in vivo experiments.

**Table 2:** Working solutions for treatments used in this study.

Treatments	Unit	Working Solutions (% *w/v* or *v/v*) for							
		Single Applications							Mixtures
		C6	C5	C4	C3	C2	C1		
Chili pepper, garlic, and peppermint	g/L	40	30	20	10	5	1		40
Bathing soap, laundry soap, liquid soap	g/L	1.0	0.8	0.6	0.5	0.3	0.1		1.0
Insecticidal soap (positive control), neem oil, and nimbecidine (positive control)	mL/L	0.6	0.5	0.4	0.3	0.2	0.1		0.6 or 1.0 *
Acetamectin Force^®^ (positive control)	mL/L							0.15	0.15
Distilled water and fermented carbohydrate–yeast mixture with water only (negative control)								0.0	0.0

* Insecticidal soap and neem oil dilution concentrations of 1% were used in the mixtures.

**Table 3:** Lethal concentration 50 values of the botanical and soap treatments against *P. nigronervosa* in laboratory at 96 hps.

Category	Treatment	LC_50_ Estimate (mg/mL)	se	LCL	UCL	Slope	slope_se	χ^2^	pgof_sig
Botanical	Nimbecidine	1.5	1.15	1.0	1.9	1.974	0.306	4.40	0.355
	Neem oil	10.0	1.61	5.7	1473.2	1.015	0.430	1.44	0.837
	Garlic	184.6	1.18	128.3	255.7	1.203	0.236	6.71	0.152
	Chili pepper	381.6	1.46	210.2	1513.4	0.691	0.202	5.40	0.249
	Peppermint	5259.8	8.39	729.6	3.6E-05	0.547	0.338	0.65	0.958
Soap	Insecticidal soap	3.6	1.14	2.5	4.8	1.322	0.378	2.77	0.597
	Bathing soap	22.0	1.98	10.4	9.2E+08	0.880	0.417	3.52	0.475
	Bar soap	32.0	2.67	11.9	0.0	0.770	0.417	2.28	0.685
	Liquid soap	154.0	17.12	17.7	4.0E-02	0.556	0.469	0.59	0.964

se = standard error of the LC_50_ estimate, LCL = lower 95% confidence limit for LC_50_, UCL = upper 95% confidence limit for LC_50_, slope = slope of the concentration–mortality curve, slope_se = standard error of the slope estimate, χ^2^ = goodness-of-fit chi-square statistic, pgof_sig = *p*-value for goodness of fit where *p* > 0.05 indicates a good model fit.

**Table 4:** Percentage mortality of *P. nigronervosa* for highest concentration (C6) of single application of botanicals and soaps over time in vitro. ‘hps’ denotes hours post-spray.

Category	Treatment	24 hps	48 hps	72 hps	96 hps
Botanical	Nimbecidine	87.2 ± 4.1 ^a^	88.1 ± 4.4 ^a^	89.6 ± 3.7 ^ab^	91.6 ± 3.1 ^ab^
	Fermented chili pepper	42.7 ± 3.3 ^bc^	58.5 ± 5.1 ^bc^	69.9 ± 5.9 ^bcd^	76.6 ± 6.2 ^abc^
	Garlic	33.3 ± 8.8 ^cd^	49.9 ± 8.0 ^bcd^	72.5 ± 8.5 ^bc^	73.7 ± 9.2 ^abcd^
	Chili pepper	34.4 ± 9.2 ^cd^	43.7 ± 5.3 ^bcde^	51.7 ± 9.0 ^cdef^	60.0 ± 9.8 ^cdef^
	Fermented garlic	6.7 ± 1.3 ^f^	16.8 ± 1.0 ^fgh^	34.6 ± 2.2 ^fg^	51.7 ± 4.4 ^cdefg^
	Neem oil	32.5 ± 4.7 ^cd^	37.8 ± 4.0 ^cdef^	43.0 ± 2.8 ^efg^	46.3 ± 4.1 ^defg^
	Fermented peppermint	10.3 ± 3.2 ^ef^	17.0 ± 3.7 ^fgh^	24.8 ± 4.4 ^gh^	34.2 ± 4.8 ^fg^
	Peppermint	15.4 ± 2.1 ^def^	23.3 ± 3.1 ^efg^	27.2 ± 4.2 ^fgh^	29.6 ± 4.0 ^gh^
Soap	Insecticidal soap	62.6 ± 5.3 ^b^	63.0 ± 5.6 ^b^	63.4 ± 4.8 ^cde^	65.4 ± 5.1 ^bcde^
	Bathing soap	35.0 ± 4.4 ^cd^	43.2 ± 7.9 ^bcde^	45.2 ± 9.6 ^defg^	45.4 ± 10.0 ^efg^
	Bar soap	31.1 ± 2.6 ^cde^	32.3 ± 2.7 ^def^	37.5 ± 3.7 ^fg^	40.5 ± 3.2 ^efg^
	Liquid soap	18.7 ± 4.1 ^def^	20.2 ± 4.1 ^fgh^	24.4 ± 4.2 ^gh^	28.6 ± 4.1 ^ghi^
Control	Acetamectin Force	98.4 ± 0.4 ^a^	100.0 ± 0.0 ^a^	100.0 ± 0.0 ^a^	100.0 ± 0.0 ^a^
	Fermented carbohydrate–yeast mixture with water only	1.4 ± 0.9 ^f^	2.0 ± 1.4 ^gh^	3.4 ± 2.2 ^h^	4.6 ± 1.8 ^hi^
	Water control	0.2 ± 0.1 ^f^	0.6 ± 0.2 ^h^	1.6 ± 0.4 ^h^	2.1 ± 0.4 ^i^

^a–i^ Within columns, means (± standard error (SE) followed by different letters significantly differ at 5% level of Tukey’s HSD test.

**Table 5:** Percentage mortality of *P. nigronervosa* when treated with mixtures of non-fermented or fermented botanicals with soaps over time in vitro. ‘hps’ denotes hours post-spray.

Category	Treatment	24 hps	48 hps	72 hps	96 hps
Insecticidal soap based	Garlic + insecticidal soap	90.8 ± 0.4 ^a^	98.3 ± 1.7 ^a^	100.0 ± 0.0 ^a^	100.0 ± 0.0 ^a^
	Nimbecidine + insecticidal soap	93.8 ± 2.0 ^a^	100.0 ± 0.0 ^a^	100.0 ± 0.0 ^a^	100.0 ± 0.0 ^a^
	Fermented peppermint + insecticidal soap	89.2 ± 4.2 ^a^	93.7 ± 4.2 ^a^	97.4 ± 2.6 ^ab^	98.2 ± 1.8 ^ab^
	Fermented garlic + insecticidal soap	79.4 ± 5.5 ^ab^	91.4 ± 5.0 ^a^	93.4 ± 5.4 ^abc^	96.9 ± 3.1 ^ab^
	Fermented chili pepper + insecticidal soap	85.4 ± 5.0 ^a^	92.9 ± 3.0 ^a^	93.4 ± 4.1 ^abc^	93.8 ± 2.9 ^abc^
	Neem oil + insecticidal soap	87.6 ± 3.0 ^a^	87.8 ± 4.2 ^ab^	89.3 ± 4.6 ^abcd^	92.9 ± 4.6 ^abc^
	Chili pepper + insecticidal soap	71.0 ± 6.9 ^ab^	73.4 ± 7.4 ^abc^	76.9 ± 6.9 ^abcdef^	77.9 ± 7.2 ^abcde^
	Peppermint + insecticidal soap	53.1 ± 2.4 ^bc^	59.3 ± 3.7 ^bcd^	61.3 ± 2.6 ^defg^	65.3 ± 2.0 ^def^
Bar soap based	Nimbecidine + bar soap	80.8 ± 4.4 ^ab^	83.0 ± 4.4 ^ab^	83.6 ± 3.2 ^abcde^	85.5 ± 3.6 ^abcd^
	Neem oil + bar soap	51.7 ± 9.6 ^bc^	60.6 ± 6.5 ^bcd^	69.4 ± 5.0 ^bcdef^	71.8 ± 5.2 ^bcde^
	Fermented chili pepper + bar soap	53.9 ± 3.8 ^bc^	61.1 ± 6.5 ^bcd^	67.4 ± 6.6 ^cdef^	67.7 ± 6.7 ^cde^
	Garlic + bar soap	53.2 ± 6.5 ^bc^	54.3 ± 6.3 ^cd^	56.6 ± 6.0 ^efg^	57.0 ± 5.5 ^ef^
	Chili pepper + bar soap	31.1 ± 6.6 ^cde^	46.1 ± 6.2 ^cde^	51.2 ± 4.3 ^fg^	56.4 ± 5.2 ^ef^
	Fermented garlic + bar soap	40.2 ± 15.4 ^cd^	46.0 ± 13.8 ^cde^	50.5 ± 12.4 ^fg^	53.4 ± 12.5 ^ef^
	Peppermint + bar soap	40.9 ± 2.8 ^c^	44.7 ± 4.7 ^de^	49.4 ± 5.6 ^fg^	51.0 ± 4.2 ^ef^
	Fermented peppermint + bar soap	10.8 ± 3.8 ^de^	24.6 ± 6.2 ^ef^	37.5 ± 10.0 ^g^	39.0 ± 10.1 ^f^
Control	Acetamectin Force	99.9 ± 0.1 ^a^	100.0 ± 0.0 ^a^	100.0 ± 0.0 ^a^	100.0 ± 0.0 ^a^
	Water control	2.1 ± 0.3 ^e^	2.9 ± 0.3 ^f^	3.3 ± 0.4 ^h^	3.3 ± 0.4 ^g^

^a–h^ Within columns, means (±SE) followed by different letters differ significantly at 5% level of Tukey’s HSD test.

**Table 6:** *Pentalonia nigronervosa* aphid population when treated with mixtures of biorationals in vivo, assessed weekly from onset to closure of experiments.

Category	Treatment	Time in Weeks (W)								
		W0	W1	W2	W3	W4	W5	W6	W7	W8
Insecticidal soap based	Neem oil + insecticidal soap	80.1 ± 26.1 ^a^	8.6 ± 1.3 ^c^	21.7 ± 6.2 ^b^	6.0 ± 2.1 ^b^	9.3 ± 3.7 ^b^	4.5 ± 1.7 ^b^	6.1 ± 1.8 ^b^	2.2 ± 1.0 ^b^	1.0 ± 0.4 ^b^
	Garlic + insecticidal soap	79.4 ± 17.4 ^a^	6.9 ± 2.0 ^c^	12.7 ± 5.6 ^b^	3.5 ± 0.9 ^b^	6.8 ± 1.9 ^b^	5.8 ± 2.0 ^b^	15.1 ± 6.5 ^b^	4.2 ± 0.7 ^b^	4.4 ± 1.0 ^b^
	Fermented garlic + insecticidal soap	78.4 ± 31.9 ^a^	10.2 ± 5.8 ^bc^	15.6 ± 10.7 ^b^	4.6 ± 3.0 ^b^	9.1 ± 4.7 ^b^	8.2 ± 6.5 ^b^	14.7 ± 12.0 ^b^	3.4 ± 2.2 ^b^	7.8 ± 4.5 ^b^
	Fermented peppermint + insecticidal soap	75.1 ± 9.4 ^a^	18.5 ± 4.8 ^bc^	23.7 ± 4.9 ^b^	4.6 ± 1.0 ^b^	8.1 ± 2.7 ^b^	5.5 ± 2.0 ^b^	9.7 ± 3.9 ^b^	9.2 ± 2.8 ^b^	11.6 ± 3.7 ^b^
	Fermented chili pepper + insecticidal soap	76.2 ± 26.5 ^a^	17.1 ± 7.7 ^bc^	27.7 ± 12.1 ^b^	12.3 ± 3.6 ^b^	23.9 ± 8.4 ^b^	12.8 ± 4.0 ^b^	31.0 ± 13.2 ^b^	11.7 ± 3.6 ^b^	21.8 ± 7.4 ^b^
	Peppermint + insecticidal soap	77.7 ± 11.6 ^a^	48.2 ± 16.5 ^bc^	68.9 ± 18.5 ^b^	55.6 ± 19.7 ^b^	62.8 ± 18.6 ^b^	44.9 ± 12.0 ^b^	50.0 ± 13.9 ^b^	20.0 ± 1.5 ^b^	30.6 ± 1.9 ^b^
	Chili pepper + insecticidal soap	82.4 ± 19.4 ^a^	26.3 ± 7.4 ^bc^	40.0 ± 9.5 ^b^	17.9 ± 5.0 ^b^	17.6 ± 3.5 ^b^	16.8 ± 3.4 ^b^	36.8 ± 7.9 ^b^	25.8 ± 6.5 ^b^	43.0 ± 13.5 ^b^
Bar soap based	Neem oil + bar soap	73.0 ± 24.5 ^a^	38.7 ± 15.6 ^bc^	40.4 ± 17.7 ^b^	6.7 ± 3.0 ^b^	6.9 ± 3.2 ^b^	6.8 ± 2.8 ^b^	12.0 ± 7.3 ^b^	8.5 ± 5.3 ^b^	15.8 ± 9.0 ^b^
	Nimbecidine^®^ + bar soap	78.0 ± 19.1 ^a^	39.9 ± 10.9 ^bc^	60.8 ± 13.1 ^b^	29.5 ± 9.9 ^b^	40.5 ± 14.2 ^b^	21.5 ± 6.6 ^b^	38.5 ± 9.1 ^b^	18.0 ± 5.8 ^b^	26.2 ± 9.2 ^b^
	Fermented chili pepper + bar soap	68.2 ± 9.0 ^a^	39.2 ± 11.4 ^bc^	60.2 ± 16.0 ^b^	30.1 ± 10.1 ^b^	41.2 ± 14.3 ^b^	26.0 ± 5.2 ^b^	45.8 ± 9.1 ^b^	25.7 ± 5.3 ^b^	36.2 ± 8.9 ^b^
	Garlic + bar soap	73.6 ± 17.4 ^a^	65.5 ± 13.4 ^ab^	76.8 ± 15.5 ^b^	51.8 ± 20.1 ^b^	58.3 ± 21.3 ^b^	38.7 ± 6.5 ^b^	52.8 ± 6.4 ^b^	37.2 ± 5.2 ^b^	54.2 ± 3.8 ^b^
Control	Water control	77.1 ± 17.6 ^a^	120.4 ± 20.4 ^a^	146.1 ± 21.0 ^a^	185.0 ± 19.3 ^a^	185.8 ± 21.2 ^a^	199.0 ± 24.9 ^a^	206.2 ± 29.4 ^a^	211.1 ± 31.0 ^a^	219.0 ± 29.5 ^a^

^a–c^ Within columns, means (±SE) followed by different letters differ significantly at 5% level Tukey’s HSD test. Spray applications were performed bi-weekly during weeks W0 (Spray 1), W2 (Spray 2), W4 (Spray 3), and W6 (Spray 4) of experimentation.

## Data Availability

The data underlying this article will be shared on reasonable request to the corresponding author.

## Supplementary Materials

The following supporting information can be downloaded at: https://www.mdpi.com/article/10.3390/insects17010023/s1, Figure S1: Mortality of *Pentalonia nigronervosa* at 24 up to 96 hours after treatment with higher concentrations of single applications of non-fermented botanicals and soaps in vitro; Figure S2: Aphid abundance after treatment with mixtures of biorationals in vivo, assessed weekly from onset to closure of trials. Aphid abundance was scored on a scale of 0–9 modified from Biale et al. (2017) [65]; where 0 = no visible aphids, 1 = 1 aphid, 2 = 2–5 aphids, 3 = 6–20 aphids, 4 = 21–100 aphids, 5 = 101–200 aphids, 6 = 201–300 aphids, 7 = 301–400 aphids, 8 = 401–500 aphids and 9 = more than 500 aphids; Table S1: Repeated measures analysis of variance for aphid population and mortality for the in vitro and in vivo studies conducted at National Agricultural Research Laboratory, Kawanda; Table S2: Proportion of living *Pentalonia nigronervosa* aphids (%) when treated with mixtures of biorationals in vivo, assessed weekly from onset to closure of experiments; Table S3: Proportion of dead *Pentalonia nigronervosa* aphids (%) of assessed bi-weekly, 36 hours after each spray and phytotoxicity due to biorational treatment mixtures in vivo; Table S4: Cost and risk assessment for the biorational treatments used during in vitro single applications and in vivo mixtures.
