# Supplementary material for: Screening for the Efficacy of Botanicals and Soaps in Controlling the Banana Aphid Pentalonia nigronervosa (Hemiptera: Aphididae) Under Laboratory and Screenhouse Conditions
Source: Insects. 2025 Dec 23;17(1):23. doi: 10.3390/insects17010023 (PMC12841634; doi:10.3390/insects17010023)
Supplement: Supplementary file 1 [file insects-17-00023-s001.zip › Supplementary figures.pdf]

Supplementary figures

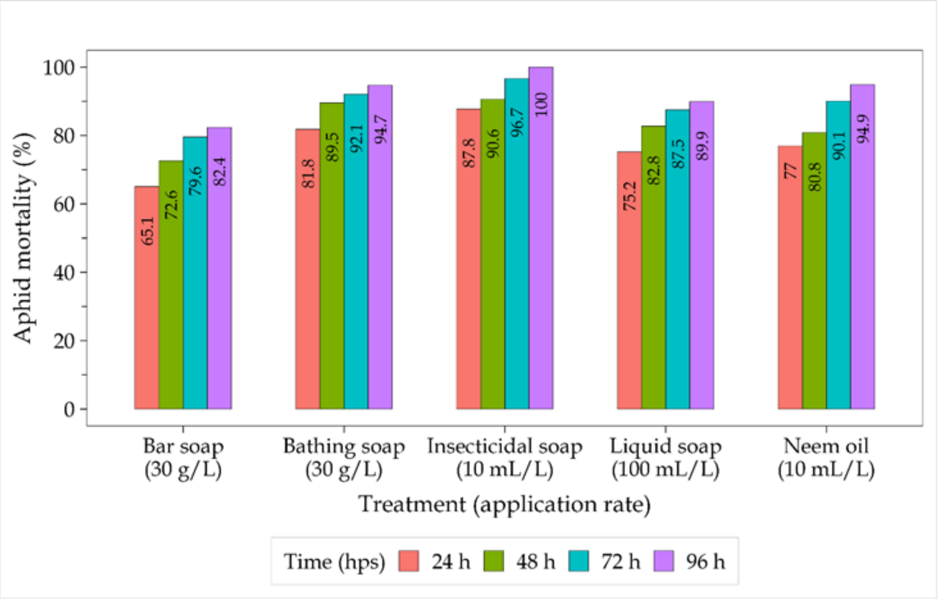

Figure S1. Mortality of *Pentalonia nigronervosa* at 24 up to 96 hours after treatment with higher concentrations of single applications of non-fermented botanicals and soaps in vitro

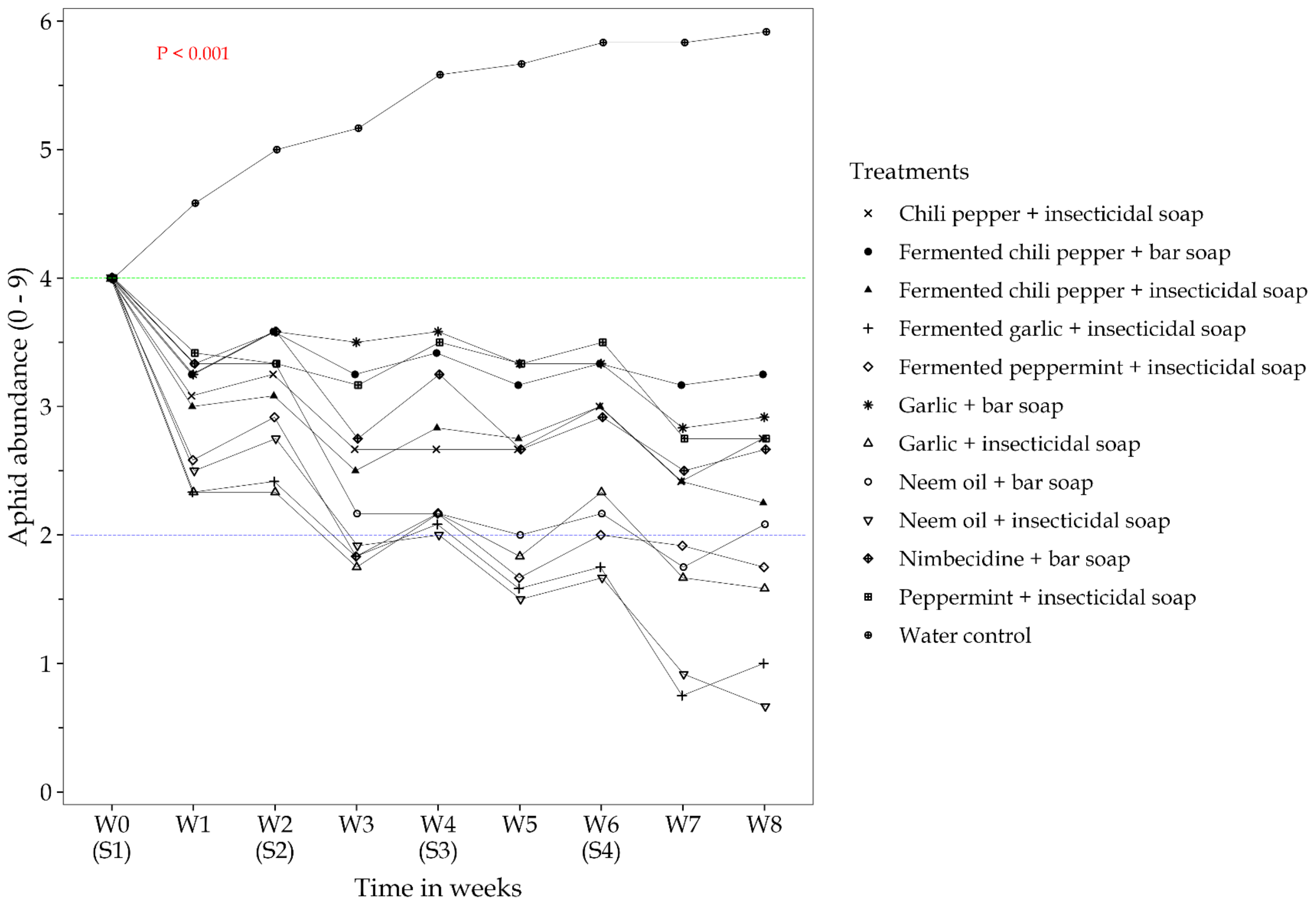

Figure S2: Aphid abundance after treatment with mixtures of biorationals in vivo, assessed weekly from onset to closure of trials. Aphid abundance was scored on a scale of 0 – 9 modified from Biale et al. (2017); where 0 = no visible aphids, 1 = 1 aphid, 2 = 2–5 aphids, 3 = 6–20 aphids, 4 = 21–100 aphids, 5 = 101–200 aphids, 6 = 201–300 aphids, 7 = 301–400 aphids, 8 = 401–500 aphids and 9 = more than 500 aphids.
