# Supplementary material for: Screening for the Efficacy of Botanicals and Soaps in Controlling the Banana Aphid Pentalonia nigronervosa (Hemiptera: Aphididae) Under Laboratory and Screenhouse Conditions
Source: Insects. 2025 Dec 23;17(1):23. doi: 10.3390/insects17010023 (PMC12841634; doi:10.3390/insects17010023)
Supplement: Supplementary file 1 [file insects-17-00023-s001.zip › Supplementary tables.pdf]

Supplementary tables

Table S1: Repeated measures analysis of variance for aphid population and mortality for the in vitro and in vivo studies conducted at National Agricultural Research Laboratory, Kawanda.

**In vitro: Single applications at different concentrations**						
**Stratum**	**Source of variation**	**df**	**sum sq**	**mean sq**	**F-statistic**	**p-value**
Replication	Residuals	5	13,323	2,665		
Replication × hps	Hours post spray (hps)	3	21,954	7,318	107.9	< 0.001
	Residuals	15	1,017	68		
	Treatment	8	301,183	37,648	189.0	< 0.001
	Concentration	5	143,690	28,738	144.3	< 0.001
	Treatment × concentration	40	42,043	1,051	5.3	< 0.001
	Treatment × hps	24	5,459	227	1.1	0.289
	Concentration × hps	15	966	64	0.3	0.993
	Treatment × concentration × hps	120	5,589	47	0.23	1.000
	Residuals	1060	211,120	199		
Total		1295	746,343			
**In vitro: Single applications at highest concentration (C6)**						
Replication	Residuals	5	755	151		
Replication × hps	Hours post spray (hps)	3	13,368	4,456	103.0	< 0.001
	Residuals	15	649	43		
	Treatment	14	269,666	19,262	127.4	< 0.001
	Treatment × hps	42	11,234	267	1.77	0.0039
	Residuals	280	42,336	151		
Total		359	338,006			
**In vitro: Mixed applications**						
Replication	Residuals	5	3,057	611		
Replication × hps	Hours post spray (hps)	3	7,435	2,478	86.0	< 0.001
	Residuals	15	432	29		
	Treatment	17	306,451	18,027	95.9	< 0.001
	Treatment × hps	51	4,091	80	0.43	0.9998
	Residuals	340	63,912	188		
Total		431	385,379			
**In vivo: Living aphid populations**						
Replication	Residuals	3	29,524	9,841		
Replication × week	Weeks	8	75,065	9,383	13.9	< 0.001
	Residuals	24	16,184	674		
	Treatment	11	738,413	67,128	124.0	< 0.001
	Treatment × weeks	88	149,289	1,696	3.13	< 0.001
	Residuals	297	160,748	541		
Total		431	1,169,223			
**In vivo: Proportion of living aphids**						
Replication	Residuals	3	12,205	4,068		
Replication × week	Weeks	8	93,388	11,673	28.7	< 0.001
	Residuals	24	9,753	406		
	Treatment	11	1,543,649	140,332	150.5	< 0.001
	Treatment × weeks	88	305,321	3,470	3.72	< 0.001
	Residuals	297	277,004	933		
Total		431	2,241,320			
**In vivo: Aphid percentage mortality**						
Replication	Residuals	3	2,892	964		
Replication × spray	Spray weeks	3	249	83	0.273	0.844
	Residuals	9	2,741	305		
	Treatment	11	724,407	65,855	111.2	< 0.001
	Treatment × spray weeks	33	81,816	2,479	4.2	< 0.001
	Residuals	132	78,198	592		
Total		191	890,303			

Df = degrees of freedom, sum sq = sum of squares, mean sq = mean sum of squares, F-statistic = F statistical value, p-value = F probability value

Table S2: Proportion of living *Pentalonia nigronervosa* aphids (%) when treated with mixtures of biorationals in vivo, assessed weekly from onset to closure of experiments.

Category	Treatment	Time in weeks (W)							
		W1	**W2**	W3	**W4**	W5	**W6**	W7	W8
Insecticidal soap based	Neem oil + insecticidal soap	18.7 ± 4.7^{e}	28.0 ± 5.2^{c}	7.3 ± 1.1^{b}	10.2 ± 1.4^{b}	5.0 ± 1.6^{c}	6.5 ± 1.5^{b}	1.2 ± 0.5^{b}	0.6 ± 0.2^{b}
	Fermented garlic + insecticidal soap	23.2 ± 5.0^{de}	33.0 ± 13.1^{bc}	14.0 ± 7.5^{b}	17.7 ± 6.8^{b}	5.8 ± 2.5^{bc}	8.8 ± 4.7^{b}	1.4 ± 0.9^{b}	3.8 ± 2.2^{b}
	Garlic + insecticidal soap	19.4 ± 6.3^{e}	31.6 ± 14.0^{c}	14.7 ± 5.0^{b}	52.7 ± 22.4^{b}	30.1 ± 15.4^{bc}	47.5 ± 16.3^{b}	20.1 ± 4.8^{b}	21.2 ± 5.8^{b}
	Fermented chili pepper + insecticidal soap	28.2 ± 3.3^{cde}	56.4 ± 17.4^{bc}	36.6 ± 10.8^{b}	54.7 ± 9.4^{b}	30.1 ± 4.0^{bc}	48.0 ± 9.1^{b}	20.9 ± 4.4^{b}	28.4 ± 6.5^{b}
	Chili pepper + insecticidal soap	36.5 ± 9.3^{cde}	47.9 ± 9.1^{bc}	23.6 ± 7.3^{b}	21.1 ± 3.9^{b}	21.4 ± 3.8^{bc}	47.1 ± 11.7^{b}	23.7 ± 5.0^{b}	36.3 ± 6.2^{b}
	Peppermint + insecticidal soap	60.7 ± 13.6^{bc}	80.3 ± 22.7^{bc}	66.2 ± 26.9^{b}	83.2 ± 27.7^{b}	74.9 ± 22.1^{b}	77.5 ± 21.4^{b}	34.4 ± 10.1^{b}	37.2 ± 11.2^{b}
	Fermented peppermint + insecticidal soap	28.5 ± 1.9^{cde}	35.4 ± 5.4^{bc}	12.9 ± 3.1^{b}	19.9 ± 5.4^{b}	27.7 ± 16.9^{bc}	41.3 ± 20.6^{b}	48.0 ± 32.7^{b}	46.0 ± 33.2^{b}
Bar soap based	Neem oil + bar soap	52.8 ± 3.4^{bcde}	53.8 ± 6.1^{bc}	12.4 ± 2.6^{b}	11.6 ± 2.9^{b}	10.5 ± 3.5^{bc}	14.1 ± 6.2^{b}	9.7 ± 4.3^{b}	16.2 ± 5.8^{b}
	Nimbecidine® + bar soap	49.6 ± 2.1^{bcde}	85.1 ± 2.2^{bc}	49.1 ± 15.9^{b}	60.3 ± 17.9^{b}	29.9 ± 5.8^{bc}	57.8 ± 11.8^{b}	36.0 ± 13.3^{b}	48.5 ± 18.1^{b}
	Fermented chili pepper + bar soap	57.1 ± 13.6^{bcd}	86.5 ± 21.4^{bc}	51.1 ± 16.3^{b}	68.8 ± 24.1^{b}	48.6 ± 9.4^{bc}	78.5 ± 11.6^{b}	44.3 ± 9.4^{b}	62.4 ± 18.6^{b}
	Garlic + bar soap	76.3 ± 4.3^{b}	97.5 ± 7.4^{b}	82.1 ± 42.4^{b}	92.4 ± 45.0^{b}	54.9 ± 12.0^{bc}	68.0 ± 16.6^{b}	55.3 ± 19.3^{b}	73.7 ± 13.3^{b}
Control	Water control	176.4 ± 7.6^{a}	220.2 ± 13.4^{a}	278.8 ± 26.3^{a}	278.7 ± 23.2^{a}	291.7 ± 32.4^{a}	301.9 ± 29.9^{a}	306.2 ± 33.9^{a}	322.2 ± 52.4^{a}

[a,b,c,d,e] Means (± SE) followed by different letters within columns are significantly different at 5% Tukey HSD test. Spray applications were done bi-weekly (in bold) at weeks W0 (Spray 1), W2 (Spray 2), W4 (Spray 3) and W6 (Spray 4) of experimentation

Table S3. Proportion of dead *Pentalonia nigronervosa* aphids (%) of assessed bi-weekly, 36 hours after each spray and phytotoxicity due to biorational treatment mixtures in vivo.

Treatment	Aphid mortality (%) after each spray (Counts 36 h after spray)				Phyto-toxicity (%)
	**S1 at week 0**	**S2 at week 2**	**S3 at week 4**	**S4 at week 6**	
Neem oil + insecticidal soap	80.3 ± 5.5[a]	93.4 ± 0.8[a]	95.3 ± 1.9[a]	97.8 ± 1.1[a]	0.0 ± 0.0[c]
Fermented garlic + insecticidal soap	79.5 ± 5.8[a]	87.4 ± 7.9[a]	87.1 ± 7.8[ab]	97.2 ± 1.0[a]	3.5 ± 0.4[ab]
Neem oil + bar soap	46.4 ± 4.5[bc]	79.6 ± 3.7[a]	91.7 ± 2.6[ab]	89.8 ± 4.7[a]	0.0 ± 0.0[c]
Fermented chili pepper + insecticidal soap	78.4 ± 7.8[a]	71.1 ± 10.4[a]	76.4 ± 6.7[ab]	82.2 ± 3.2[a]	6.0 ± 0.1[a]
Garlic + insecticidal soap	80.4 ± 1.2[a]	89.7 ± 2.6[a]	81.6 ± 7.4[ab]	80.9 ± 5.3[a]	2.8 ± 0.2[bc]
Chili pepper + insecticidal soap	69.5 ± 2.6[ab]	79.1 ± 6.2[a]	82.0 ± 5.5[ab]	75.9 ± 3.7[a]	0.0 ± 0.0[c]
Nimbecidine + bar soap	56.1 ± 3.9[abc]	63.4 ± 11.2[a]	77.2 ± 2.4[ab]	72.4 ± 10.0[a]	0.0 ± 0.0[c]
Peppermint + insecticidal soap	36.3 ± 8.5[c]	40.7 ± 23.5[a]	41.8 ± 18.0[b]	66.3 ± 12.3[a]	0.0 ± 0.0[c]
Fermented chili pepper + bar soap	38.8 ± 10.8[c]	61.2 ± 13.7[a]	68.0 ± 5.6[ab]	59.5 ± 8.5[a]	0.5 ± 0.1[c]
Garlic + bar soap	30.3 ± 7.8[c]	39.0 ± 19.6[a]	47.3 ± 9.7[ab]	51.1 ± 22.4[a]	0.5 ± 0.1[c]
Fermented peppermint + insecticidal soap	74.7 ± 2.3[ab]	87.8 ± 2.6[a]	85.5 ± 5.6[ab]	48.5 ± 37.6[a]	0.0 ± 0.0[c]
Water control	-38.9 ± 3.3[d]	-156.6 ± 20.8[b]	-178.8 ± 25.8[c]	-204.9 ± 31.1[b]	0.0 ± 0.0[c]

Each value is a mean ± SE of four replicates; [a,b,c,d,e] means followed by different letters within the same column are significantly different at 5% Tukey HSD test; S1, S2, S3 and S4 denote spray 1, 2, 3 and spray 4. Negative mortality or decrease in mortality is an indicator of aphid natality

Table S4: Cost and risk assessment for the biorational treatments used during in vitro single applications and in vivo mixtures

Treatment	Market cost for 1L biorational ($)	Cost of preparing 1L of biorational working solution ($)	Cost of spraying a mat of 4 plants i.e. 1.8 L of diluted spray ($)	Ease of working solution preparation	Possible environmental risk at recommended dosage
Fermented chili pepper + insecticidal soap	14.94	0.705	1.269	***	***
Fermented garlic + insecticidal soap	14.94	0.705	1.269	**	**
Fermented peppermint + insecticidal soap	14.94	0.705	1.269	**	**
Chili pepper + insecticidal soap	14.86	0.676	1.216	**	***
Garlic + insecticidal soap	14.86	0.676	1.216	**	**
Peppermint + insecticidal soap	14.86	0.676	1.216	**	**
Fermented chili pepper + bar soap	3.18	0.587	1.057	***	***
Fermented chili pepper	1.42	0.570	1.026	***	***
Fermented garlic	1.42	0.570	1.026	***	*
Fermented peppermint	1.42	0.570	1.026	**	*
Garlic + bar soap	3.11	0.558	1.005	**	**
Chili pepper	1.35	0.541	0.973	***	***
Garlic	1.35	0.541	0.973	**	*
Peppermint	1.35	0.541	0.973	**	*
Neem oil + insecticidal soap	27.03	0.270	0.486	*	**
Neem oil + bar soap	15.27	0.153	0.275	*	**
Insecticidal soap	13.51	0.135	0.243	*	**
Neem oil	13.51	0.135	0.243	*	*
Nimbecidine + bar soap	11.22	0.074	0.134	**	**
Nimbecidine	9.46	0.057	0.102	*	**
Bathing soap	4.05	0.041	0.073	*	**
Bar soap	1.76	0.018	0.032	*	***
Liquid soap	1.35	0.014	0.024	*	**
Acetamectin Force	8.11	0.012	0.022	****	****
Fermented mixture with water only	0.07			*	None
Distilled water	-				
Sugar	0.03				
Glucose	0.04				

Where ease of application rating * = Super easy, ** = Very easy, *** = Easy, **** = Moderate, ***** = Difficult, ****** = Very difficult; and environmental risk rating * = very low risk; ** = low risk, *** = Moderate risk, **** = High risk, ***** = very high risk.
